# Triplet Rydberg States of Aluminum Monofluoride

**DOI:** 10.1021/acs.jpca.4c00611

**Published:** 2024-03-29

**Authors:** N. Walter, M. Doppelbauer, S. Schaller, X. Liu, R. Thomas, S. Wright, B. G. Sartakov, G. Meijer

**Affiliations:** Department of Molecular Physics, Fritz-Haber-Institut der Max-Planck-Gesellschaft, Faradayweg 4-6, D-14195 Berlin, Germany

## Abstract

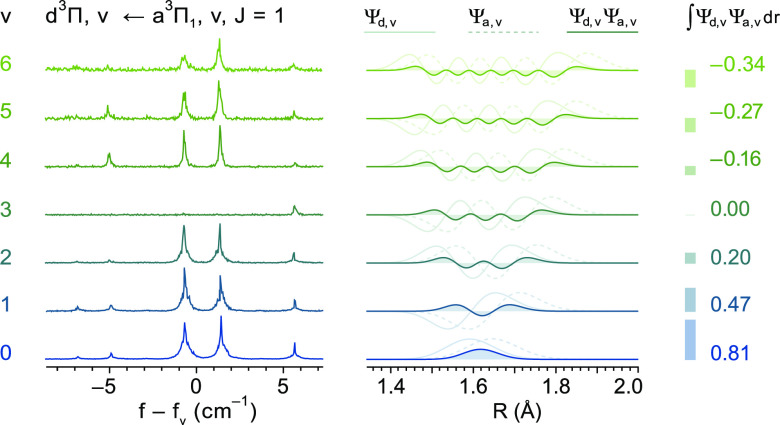

Aluminum monofluoride (AlF) is a suitable molecule for laser cooling and trapping. Such experiments require extensive spectroscopic characterization of the electronic structure. Two of the theoretically predicted higher-lying triplet states of AlF, the counterparts of the well-characterized D^1^Δ and E^1^Π states, had not been experimentally identified yet. We here report on the characterization of the d^3^Π (*v* = 0–6) and e^3^Δ (*v* = 0–2) states, confirming the predicted energetic ordering of these states (*J. Chem. Phys.***1988,***88,* 5715–5725), as well as of the f^3^Σ^+^ (*v* = 0–2) state. The transition intensity of the d^3^Π, *v* = 3 – a^3^Π, *v* = 3 band is negligibly small. This band gets its weak, unexpected rotational structure via intensity borrowing from the nearby e^3^Δ, *v* = 2 – a^3^Π, *v* = 3 band, made possible via spin–orbit and spin–rotation interaction between the d^3^Π and e^3^Δ states. This interaction affects the equilibrium rotational constants in both states; their deperturbed values yield equilibrium internuclear distances that are consistent with the observations. We determined the ionization potential of AlF to be 78,492(1) cm^–1^ by ionization from the d^3^Π state.

## Introduction

The first comprehensive overview of the electronic spectrum of gaseous AlF was given by Barrow, Kopp, and Malmberg in 1974.^1^ At that time, a total of nine singlet states (including the X^1^Σ^+^ ground state) and seven triplet states had been assigned to AlF. These states were ordered into a common energy scheme, and the nomenclature for some of the triplet states was revised to get a labeling in alphabetic order with increasing energy. A state that had been tentatively assigned as a ^3^Δ state,^2^ the counterpart of the well-characterized D^1^Δ state, was designated as the d^3^Δ state, but it was given a question mark. The rotational structure of this state had not been resolved and the state (originally denoted as c^3^Σ) was only characterized by the energy of its lowest two vibrational levels.^3^ In a theoretical study of the lowest six singlet and lowest five triplet states of AlF, the assignment of this state as a ^3^Δ state was substantiated, but it was predicted that there should be a ^3^Π state, the counterpart of the E^1^Π state, about 2000 cm^–1^ lower in energy.^4^ The correct ordering of the triplet manifold was thus proposed to be a^3^Π, b^3^Σ^+^, c^3^Σ^+^, d^3^Π, and e^3^Δ, and this is the designation we will follow from now on. Of these, the lowest three states have been well characterized,^5−9^ but the d^3^Π state has never been reported upon and the rotational and fine-structure of the e^3^Δ state, needed for its unambiguous characterization, has not been resolved.

AlF has been of interest to spectroscopists all along: the electronic structure of AlF is similar to that of the intensively studied molecule CO, the benchmark diatomic molecule for studying perturbations.^10^ However, as AlF is heavier, its electronic transitions are in the more accessible region of the spectrum. When it became clear that the various properties of AlF make it an ideal candidate molecule for laser cooling and trapping experiments,^5,11,12^ e.g., because of its strong A^1^Π–X^1^Σ^+^ transition with its highly diagonal Franck–Condon matrix,^13,14^ interest in the spectroscopic properties of AlF increased further.

In this study, we report on the missing d^3^Π state of AlF and experimentally characterize the e^3^Δ state. In the early measurements of emission and absorption spectra of hot samples, the optical transitions connecting to these triplet states might have been obscured by other bands, although it is not a priori clear why these states have not been observed earlier. We use multiple lasers to perform ionization spectroscopy in a jet-cooled molecular beam and observe the *v* = 0–6 levels of the d^3^Π state via excitation from laser-prepared, single rotational levels in the a^3^Π state and the b^3^Σ^+^ state. Starting from the a^3^Π state, the *v* = 0–2 levels of the e^3^Δ state are characterized. In the 1974 overview paper,^1^ the *v* = 0 level of the next-higher triplet state, named there the e^3^Σ^+^ state, has been reported upon as well. We characterize the *v* = 0–2 levels of this same ^3^Σ^+^ state, which we propose to refer to as the f^3^Σ^+^ state from now on. Apart from the energies of the lowest ro-vibrational levels of these three electronically excited triplet states, we determine their radiative lifetimes via time-delayed ionization. Ionization from the d^3^Π state allows us to determine the absolute energies of the *v* = 0 and *v* = 1 levels in the X^2^Σ^+^ state of the AlF^+^ cation, thereby significantly improving the accuracy of the ionization potential (IP) relative to previous measurements.^15^

## Experiment

**Figure 1:**
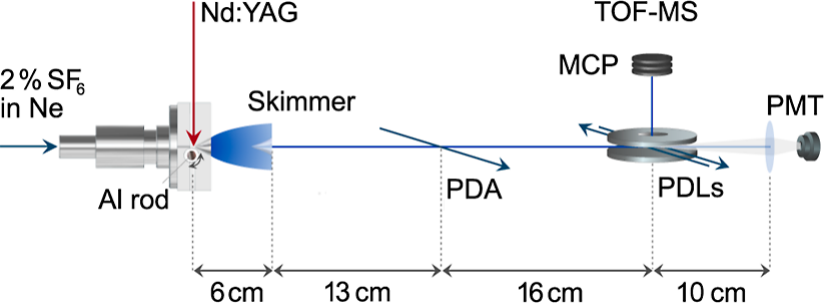
Scheme of the experimental setup. AlF molecules are produced by laser ablation of Al in a mixture of SF_6_ seeded in neon. The molecules are cooled in the expansion and subsequently prepared in selected ro-vibrational levels of the a^3^Π state by a pulsed dye amplifier (PDA). Subsequently, the molecules are excited to higher-lying triplet states and ionized by a pulsed dye laser (PDL). The AlF^+^ ion signal is detected in a time-of-flight mass spectrometer (TOF-MS).

The experimental setup is shown in Figure 1 and has been described in detail before.^5,6^ In short, a gas mixture of 2% SF_6_ in neon is released into a vacuum through a pulsed solenoid valve (General Valve, Series 9) operating at 10 Hz. Close to the valve opening, a rotating aluminum rod is laser-ablated by a focused Nd:YAG laser (Continuum Minilite I, 1064 nm, 16 mJ pulse energy, 5 ns pulse duration, 0.5 mm spot diameter). The ablated aluminum atoms react efficiently with SF_6_ in a short reaction channel to form AlF molecules. These molecules are translationally and internally cooled through collisions with the carrier gas upon expanding from the channel into vacuum. Closely behind this laser ablation source, a pair of electrodes produces an electric field of 100 V/cm to deflect ions created in the laser ablation process.

After collimation of the beam of neutral species through a conically shaped skimmer (4 mm diameter opening), the AlF molecules are excited to selected rotational levels in the metastable a^3^Π state, some 19 cm downstream from the source. Excitation is performed on the Δ*v* = 0 bands of the a^3^Π, *v* ← X^1^Σ^+^, *v* transition around 367 nm. For this, we use the frequency-doubled output of a pulsed dye amplifier (PDA) operated with Pyridine 2 dye that is injection-seeded by a narrow-band, cw titanium sapphire laser (Sirah Matisse) and pumped by a frequency-doubled Nd:YAG laser (Innolas Spitlight, 532 nm). The bandwidth of the frequency-doubled PDA radiation is about 220 MHz. The PDA contains a stimulated Brillouin scattering (SBS) cell (to filter out amplified spontaneous emission) that induces a –1.98(5) GHz shift to the fundamental frequency.^6^ The wavelength of the Ti:Sa seed-laser is measured with a calibrated wavemeter (HighFinesse WS8, 10 MHz accuracy). At the experiment, the 367 nm pulse energy is about 5–10 mJ and the collimated PDA beam (5 mm diameter) intersects the molecular beam perpendicularly. Because the lifetime of the metastable state is several milliseconds,^6^ the subsequent excitation and ionization steps can take place in a separate chamber, 16 cm further downstream, where a Wiley–McLaren-type time-of-flight mass spectrometer is installed. In the field-free region between the repeller and extractor electrodes, the molecular beam is intersected with 1–300 μJ of the frequency-doubled radiation of a pulsed dye laser (PDL, Sirah Cobra Stretch), tuned around 280 nm. The spectral bandwidth of this laser is about 1.5 GHz, and its absolute frequency is recorded with a low-resolution wavelength meter (HighFinesse WS6-600, 600 MHz absolute accuracy). The pulse energy is kept to avoid saturation and power broadening of the studied transitions. The UV excitation laser beam is spatially overlapped with 7 mJ of the counterpropagating beam from a second PDL (Radiant Dyes NarrowScan, operated on Rhodamine 101 dye). The wavelength of this laser is around 620 nm, set to bring the AlF molecules above the ionization potential. The time delay between the excitation and ionization laser pulses is optimized for the maximum signal. The voltages on the electrodes of the mass spectrometer are switched on some 100 ns after the ionization laser pulse. The AlF^+^ ions are then accelerated perpendicular to the molecular and laser beams toward a microchannel plate detector. As the excitation and ionization of the AlF molecules take place under field-free conditions, this detection scheme is parity-selective. The ion signal is amplified and recorded on a computer using a fast digitizer card. The scans are controlled with home-built LabView data acquisition software.

We optimized the source conditions and the a^3^Π state preparation by detecting the a^3^Π → X^1^Σ^+^ phosphorescence signal by a photomultiplier tube (PMT; Hamamatsu R928). For this, a *f* = 50 mm fused silica imaging lens and a 367 nm bandpass filter are installed at the end of the machine to collect radiation that is emitted along the molecular beam, i.e., in the forward direction.

Our first observation of the d^3^Π state was via excitation in the 555–565 nm region from selected ro-vibrational levels in the b^3^Σ^+^ state, followed by ionization from the upper state with the same laser. In this one-color resonance-enhanced multiple-photon ionization ((1 + 1)-REMPI) scheme, the pulse energy of the laser required for efficient ionization caused significant broadening of the resonant transitions from the b^3^Σ^+^ state. Nevertheless, the spectra were sufficiently resolved for the unambiguous assignment of the upper state as a normal ^3^Π state with a value of the *A*_*v*_/*B*_*v*_ ratio of just below ten. The spectra also served for the first determination of the absolute energies of the lowest vibrational levels of this ^3^Π state. The lowest ro-vibrational level of this ^3^Π state is about 62,507 cm^–1^ above the lowest ro-vibrational level in the X^1^Σ^+^ state of AlF. This is about 600 cm^–1^ higher than the predicted energy of the d^3^Π state.^4^ Given that the a^3^Π state, the b^3^Σ^+^ state, and the c^3^Σ^+^ state have experimentally also been found to be some 740, 570, and 220 cm^–1^ higher in energy, respectively, than in these theoretical calculations,^4^ we can safely conclude that we are dealing with the sought-after d^3^Π state.

For a more detailed characterization of the d^3^Π state, we used two-color (1 + 1′)-REMPI, starting from selected laser-prepared ro-vibrational levels in the a^3^Π state. This two-color scheme enables independent optimization of the resonant excitation and ionization steps. By using the a^3^Π state as the intermediate state, both the e^3^Δ and the f^3^Σ^+^ states that are expected in close proximity to the d^3^Π state can be reached and investigated as well. By scanning the time delay between the excitation and the ionization laser, the radiative lifetime of the levels in either one of these triplet states can be measured. Moreover, by scanning the wavelength of the ionization laser, the ionization potential (IP) can be accurately determined.

In these experiments, the AlF molecules are excited on the R_2_(*N*) (*N* = 0–4) lines of the Δ*v* = 0 bands of the a^3^Π_1_, *v* – X^1^Σ^+^, *v* transition. This way, we selectively populate the *J* = *N* + 1 rotational level with (−1)^*J*^ parity in the a^3^Π_1_, *v* state. We perform these experiments starting from *v* = 0–6 in the X^1^Σ^+^ state; in the molecular beam, the population in the *v* = 6 level is about 2 orders of magnitude less than in the *v* = 0 level, and beyond that, it is challenging to obtain an adequate signal-to-noise ratio. We then scan the 280 nm UV excitation laser over a range of 20–30 cm^–1^ to map out the ro-vibrational levels in the d^3^Π, e^3^Δ, and f^3^Σ^+^ states that can be reached from this laser-prepared level in the a^3^Π state. In total, we record the absolute energies of 183 ro-vibrational levels, which are all listed in the Supporting Information.

## Theory

The general Hamiltonian to describe the energy level structure in the electronic states of AlF can be expressed as
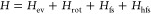
1but we can neglect the last term due to the hyperfine structure as this has not been experimentally resolved in the present study. The term *H*_ev_ describes the electronic and vibrational energy *E*_*v*_ that is commonly expressed as
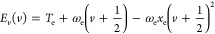
2

**Figure 2:**
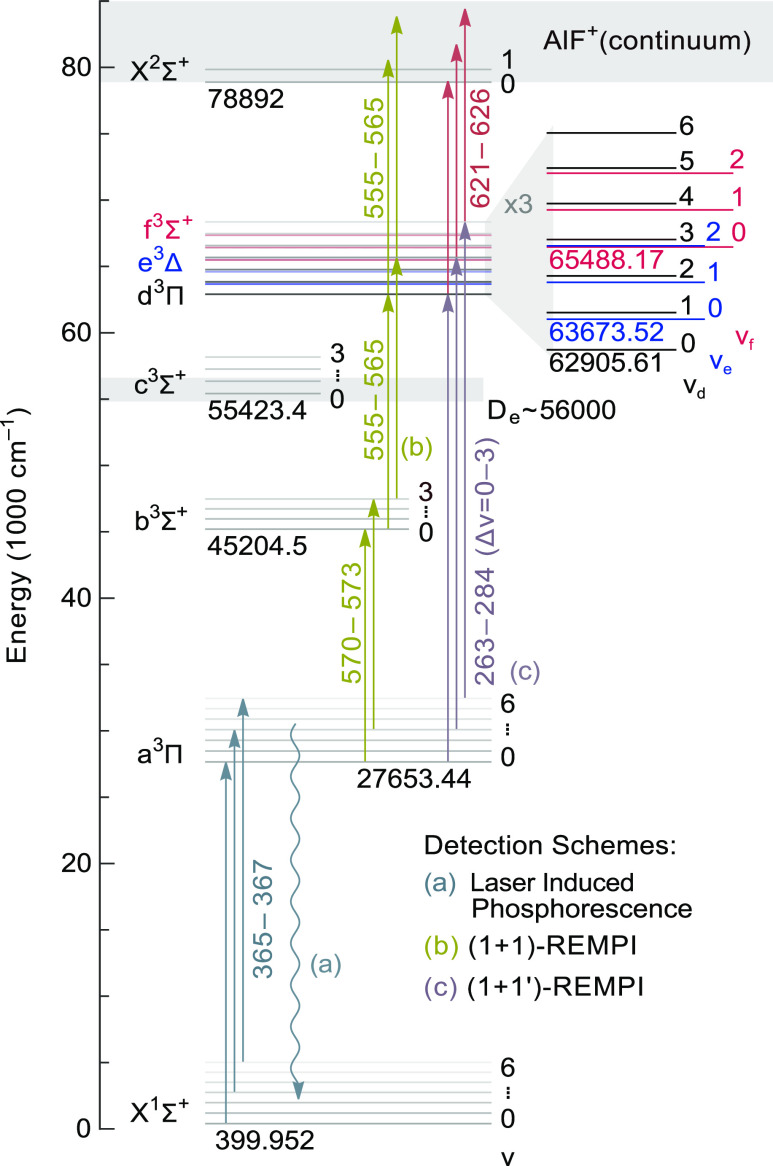
Energy level scheme of AlF, showing the ground state (X^1^Σ^+^), the lowest six triplet states (a^3^Π–f^3^Σ^+^), and the ground state of the ion (X^2^Σ^+^). For each electronic state, the lowest vibrational levels are shown. The energies given in the plot are those of the *v* = 0 level relative to the minimum of the X^1^Σ^+^ state potential.^1^ The vibrational levels shown on the expanded energy scale in the top right corner are those measured in the present study, which all lie above the dissociation energy (*D*_e_), expected between 55,000 and 56,500 cm^–1^. The vertical arrows represent laser excitation and are labeled by the relevant wavelength range in nm. Unless stated otherwise, diagonal bands (Δ*v* = 0) are used for excitation.

We follow the convention of Barrow et al.^1^ and fix *T*_e_ = 0 for the minimum of the X^1^Σ^+^ ground state potential. This puts the lowest ro-vibrational level in the X^1^Σ^+^ state at 399.952 cm^–1^, as indicated in Figure 2. The rotational part of the Hamiltonian, *H*_rot_, and the fine-structure part, *H*_fs_, are best expressed in terms of the Hund’s case (a) model for the a^3^Π state. We therefore describe both the d^3^Π state and the e^3^Δ state in terms of Hund’s case (a) as well, even though, as we will see, the ratio *A*_*v*_/*B*_*v*_ of the spin–orbit coupling constant (*A*) to the rotational constant (*B*) is much smaller in these states than in the a^3^Π state. The expression for *H*_rot_ + *H*_fs_ that we use is given by
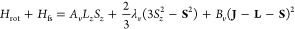
3where **L** and **S** are the total electron angular momentum and total electron spin, respectively, and where **J** is the total angular momentum, including the end-over-end rotation **N**. The projections of **L** and **S** on the internuclear axis are *L*_*z*_ and *S*_*z*_, respectively, and λ is the spin–spin interaction parameter. The subindices refer to the specific vibrational level *v* that these parameters belong to, and for the vibrational dependence of *A*_*v*_ and *B*_*v*_, we use the expressions
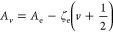
4
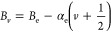
5

The f^3^Σ^+^ state is best described as Hund’s case (b), and the energy of the low rotational levels that we measured is given by *E*_rot_ = *B*_*v*_*N*(*N* + 1). Some broadening due to spin–rotation, spin–spin, or hyperfine interactions is discernible in the spectral lines to this state, but the spectral resolution is insufficient to analyze this further.

## Results

### d^3^Π State of AlF

**Figure 3:**
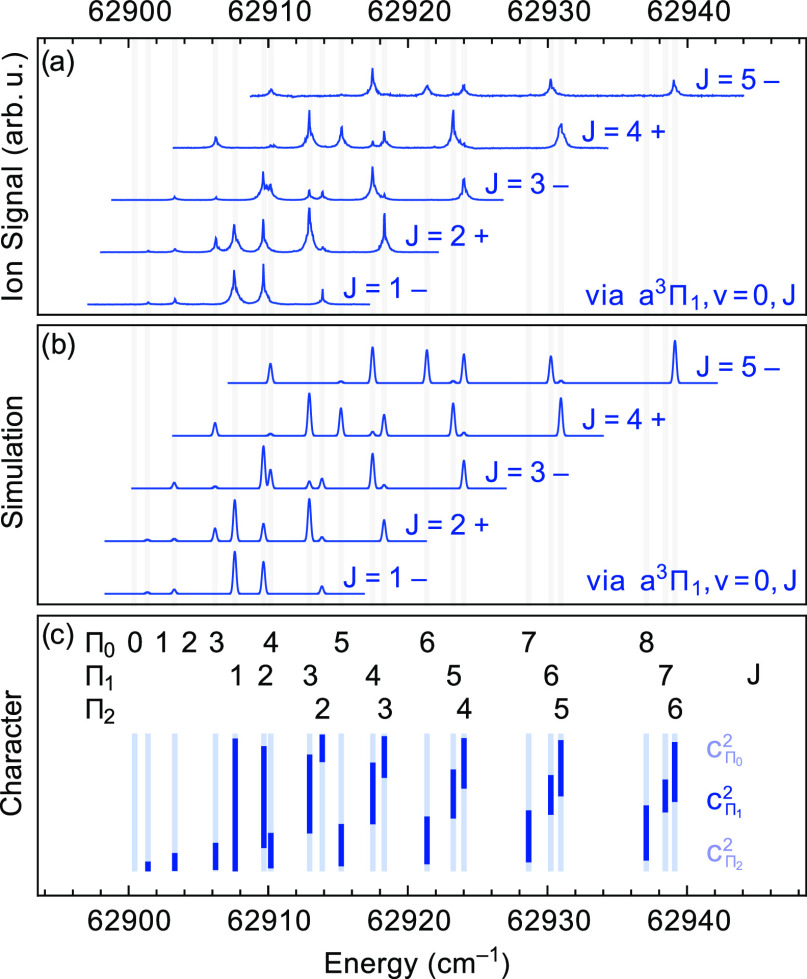
(a) Rotationally resolved spectra to the d^3^Π, *v* = 0 state recorded via different rotational levels *J* in the a^3^Π_1_, *v* = 0 state. The individual traces are normalized and vertically offset for clarity. The energy scale is relative to the minimum of the X^1^Σ^+^ state potential. (b) Simulated spectra, see text for details. (c) Vertical bars, indicating the fraction of Ω = 0, 1 (in bold), and 2 character of the *J*-levels in the d^3^Π, *v* = 0 state.

To investigate the *v* = 0–6 levels in the d^3^Π state, it is experimentally convenient to use excitation on the Δ*v* = 0 bands of the d^3^Π, *v*–a^3^Π, *v* transition as these are spectrally rather close together. Figure 3a shows the experimental spectra to the *v* = 0 level in the d^3^Π state, obtained via the *v* = 0, *J* = 1–5 levels in the a^3^Π_1_ state. The spectra are plotted on an absolute energy scale, using the known energies of the ro-vibrational levels in the X^1^Σ^+^ state^16^ and the measured energies of the two excitation lasers. The energy levels in the d^3^Π, *v* = 0 state can be readily assigned a *J* quantum number, as in Figure 3c. The vertical bars in Figure 3c indicate the fraction of Ω = 0, 1, and 2 character of each *J*-level; the fraction of Ω = 1 character is indicated in bold, and the fraction of Ω = 0 and Ω = 2 character is in lighter color above and below that, respectively. In the spectrum shown in Figure 3a, recorded via the *J* = 1 level in the a^3^Π_1_ state, the observed five peaks can be assigned, from low to high energy, to Ω = 0, *J* = 1, 2; Ω = 1, *J* = 1, 2; Ω = 2, *J* = 2, where the Ω labeling is only as good as what is given by the bars in Figure 3c. The transition to Ω = 0, *J* = 0 is very weak and visible only under excitation conditions at which the other spectral lines are strongly saturated. The same *J*-levels in the d^3^Π state are reached via excitation from rotational levels with either plus or minus parity in the a^3^Π_1_ state. As no shift between transitions from plus and minus parity levels is observed, we conclude that the Λ-doublet splitting in the d^3^Π state is considerably smaller than the width of the lines in the spectra.

From the set of measurements on the *v* = 0–6 levels of the d^3^Π state, the rotational constants, the spin–orbit coupling constants, and the spin–spin coupling constants have been determined, and these are given in Table 1. As stated earlier, the absolute energies are given relative to the minimum of the potential of the X^1^Σ^+^ state.^1^ This places the *J* = 1 level of the Ω = 1 manifold of the d^3^Π, *v* = 0 state 62,507.69 cm^–1^ above the energy of the *N* = 0, *v* = 0 level in the X^1^Σ^+^ ground state. Figure 3b shows the simulated spectra that correspond to the experimental spectra in Figure 3a. The line intensities are calculated by
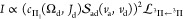
6where |*c*_Π_1__|^2^ is the amount of Π_1_ character of the reached level in the d^3^Π state, derived from the fitted spectroscopic parameters as given in Table 1 and listed in the Supporting Information.  is the overlap integral of the vibrational wave functions, and  are the according Franck–Condon factors, as given in Table 5. The Hönl–London factors are those of a ^3^Π ← ^3^Π transition, where the lower state is considered a pure Hund’s case (a), and are given by^17^
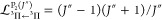
7
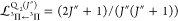
8
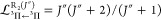
9while all others are zero.

**Table 1:** Energies *E*_*v*_, Rotational Constants *B*_*v*_, and Spin–Orbit and Spin–Spin Coupling Constants *A*_*v*_ and λ_*v*_ of the Experimentally Observed Vibrational Levels *v* in the d^3^Π State^a^

*v*	*E*_*v*_	*B*_*v*_	*A*_*v*_	λ_*v*_	σ
0	62,905.61(5)	0.591(2)	5.74(3)	–0.02(4)	0.03
1	63,839.47(6)	0.585(2)	5.75(4)	0.02(4)	0.03
2	64,763.15(6)	0.580(2)	5.80(3)	0.03(4)	0.03
3	65,676.63(6)	0.576(2)	5.81(3)	0.06(4)	0.03
4	66,579.45(3)	0.571(1)	5.84(2)	0.05(2)	0.02
5	67,471.92(9)	0.566(3)	5.84(5)	–0.02(5)	0.04
6	68,353.67(17)	0.560(6)	5.89(9)	0.03(10)	0.09

a The parameter σ is the standard deviation of the fit. All values are given in cm^–1^.

**Figure 4:**
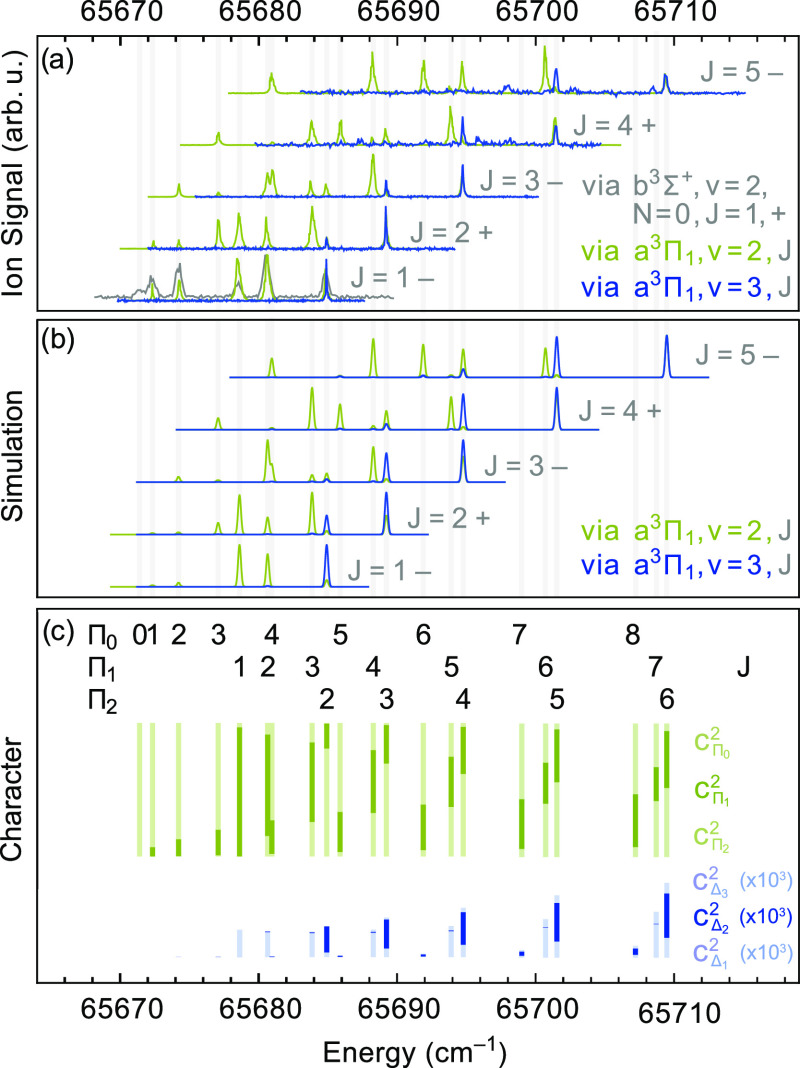
(a) Rotationally resolved spectra to the d^3^Π, *v* = 3 state recorded via different rotational levels *J* in the a^3^Π_1_, *v* = 2 state (green traces) and in the a^3^Π_1_, *v* = 3 state (blue traces). The gray trace is recorded using one-color (1 + 1)-REMPI from the b^3^Σ^+^ state. The individual traces are normalized and vertically offset for clarity. (b) Simulated spectra, see the Section on the perturbation between the e^3^Δ and d^3^Π states for details. (c) The upper row of vertical bars, in green, indicate the fraction of Ω = 0, 1 (in bold), and 2 character of the *J*-levels in the d^3^Π, *v* = 3 state. The lower row of vertical bars, in blue, indicate the fraction of Δ_1_, Δ_2_ (in bold) and Δ_3_ character of the *J*-levels in the d^3^Π, *v* = 3 state due to mixing with the e^3^Δ, *v* = 2 state.

The spectra recorded for the diagonal bands of the d^3^Π, *v*–a^3^Π, *v* transition all appear similar, apart from those belonging to the d^3^Π, *v* = 3–a^3^Π, *v* = 3 band. The latter spectra are rather weak and unexpectedly show a drastically different rotational intensity pattern, with mainly the lines to the rotational levels in the Ω = 2 manifold being detectable, as shown by the blue traces in Figure 4a. The set of spectra to the *v* = 3 level in the d^3^Π state recorded via the same set of rotational levels in the a^3^Π_1_, *v* = 2 state, i.e., on an off-diagonal band, are shown as the green traces in Figure 4a. Although these spectra are slightly saturated, they show the normal, expected line positions and intensity pattern and are very similar to the spectra shown in Figure 3. From these observations, it is concluded that the transition dipole moment of the d^3^Π, *v* = 3–a^3^Π, *v* = 3 band is near zero and that the intensity observed in this band results from intensity borrowing from a nearby transition. This intensity borrowing can occur when the d^3^Π, *v* = 3 state is perturbed by another state to which a strong transition from the a^3^Π, *v* = 3 state exists.^18^ It will be shown later that this intensity borrowing results from the weak perturbation of the d^3^Π state with the e^3^Δ state. At first, this perturbation does not appear to result in a detectable shift or distortion of the rotational energy levels in *v* = 3 or in any of the other vibrational levels of the d^3^Π state.

We measured the lifetime of several low-*J* levels in the d^3^Π, *v* = 0, 1, and 2 states and always found values of (40 ± 4) ns. This agrees well with the calculated lifetime of the d^3^Π state of 48.2 ns.^4^

### e^3^Δ state of AlF

**Figure 5:**
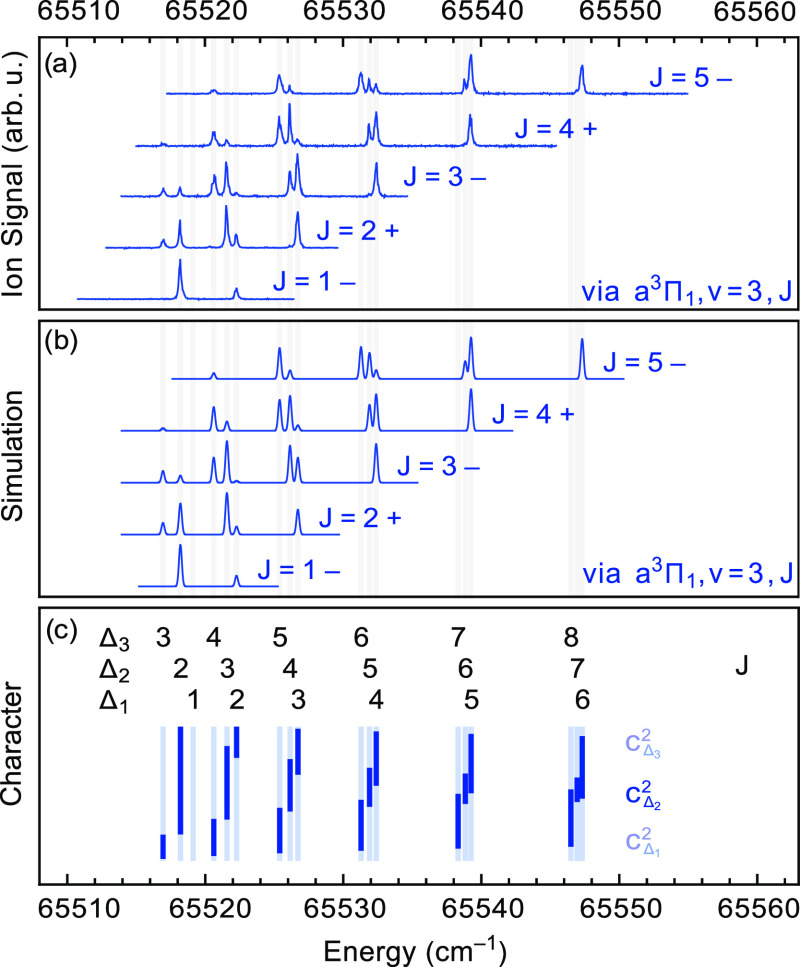
(a) Rotationally resolved spectra to the e^3^Δ, *v* = 2 state recorded via different rotational levels *J* in the a^3^Π_1_, *v* = 3 state. The individual traces are normalized and vertically offset for clarity. (b) Simulated spectra, see text for details. (c) Vertical bars, indicating the fraction of Ω = 1, 2 (in bold), and 3 character of the *J*-levels in the e^3^Δ, *v* = 2 state. Note that this state is inverted.

As mentioned in the Introduction, the lowest two vibrational levels of a state tentatively assigned as a ^3^Δ state^2^ have been observed earlier, about 36,018 and 36,948 cm^–1^, above the *v* = 0 level of the a^3^Π state.^3^ This places these levels about 160 cm^–1^ below the *v* = 1 and *v* = 2 levels, respectively, of the d^3^Π state. In order to characterize the lowest vibrational levels of the e^3^Δ state, we scanned the spectral region down to 200 cm^–1^ to the red from the diagonal d^3^Π, *v*–a^3^Π, *v* bands. The spectra of the e^3^Δ, *v* = 2–a^3^Π, *v* = 3 band, plotted on an absolute energy scale, are shown in Figure 5a. The spectra recorded via the low-*J* levels in the a^3^Π state enable an unambiguous assignment of the upper state as a ^3^Δ state with a small, negative *A*_*v*_/*B*_*v*_ ratio; that is, this state is close to Hund’s case (b) and has an inverted spin structure.

The spectra to the *v* = 0 and *v* = 1 levels of the e^3^Δ state appear similar to those shown in Figure 5a, and the rotational constants and spin–orbit coupling constants deduced from these spectra are given in Table 2. Using these values, the *J* = 2 level of the Ω = 2 manifold of the e^3^Δ, *v* = 0 state is found to be 63274.98 cm^–1^ above the energy of the *N* = 0, *v* = 0 level in the X^1^Σ^+^ ground state.

**Table 2:** Energies *E*_*v*_, Rotational Constants *B*_*v*_, and Spin–Orbit Coupling Constants *A*_*v*_ of the Experimentally Observed Vibrational Levels *v* in the e^3^Δ State^a^

*v*	*E*_*v*_	*B*_*v*_	*A*_*v*_	σ
0	63,673.52(6)	0.589(2)	–0.41(1)	0.02
1	64,601.70(6)	0.584(2)	–0.46(1)	0.02
2	65,516.7(1)	0.580(3)	–0.62(2)	0.04

a The parameter σ is the standard deviation of the fit. All values are given in cm^–1^.

It is understandable from the appearance of the energy level structure shown in Figure 5c that this upper state was mistaken for a ^3^Σ state in the early days. Only the observation of the lines to the lowest rotational levels makes the labeling with the Ω and *J* quantum numbers, as indicated in Figure 5c, possible. The vertical bars in this panel indicate the fraction of Ω = 1, 2, and 3 character of each *J*-level; the fraction of Ω = 2 character is indicated in bold and the fraction of Ω = 1 and Ω = 3 character is in lighter color below and above that, respectively.

Figure 5b shows the simulated spectra that correspond to the experimental spectra shown in the panel above. The line intensities are calculated by
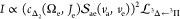
10where |*c*_Δ_2__|^2^ is the amount of Δ_2_ character of the reached level in the e^3^Δ state, derived from the fitted spectroscopic parameters, as given in Table 2 and listed in the Supporting Information.  is the overlap integral of the vibrational wave functions, and  are the according Franck–Condon factors, as given in Table 5. The Hönl–London factors are those of a ^3^Δ ← ^3^Π transition, where the lower state is considered a pure Hund’s case (a), and are given by^19^
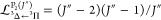
11
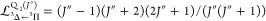
12
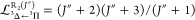
13while all others are zero. The experimental spectra are seen to be reproduced well by the simulations. The e^3^Δ_1_, *J* = 1 level cannot be reached from the a^3^Π_1_, *J* = 1 level as the Hönl–London factors for ^3^Δ_Ω_–^3^Π_1_ transitions are only nonzero for Ω = 2. We can detect this level by exciting the ground state molecules to the a^3^Π_0_, *J* = 0 level on the P_1_(1) transition before driving the e^3^Δ ← a^3^Π_0_ transition.

The intensities of the strong lines in the spectra of the e^3^Δ, *v* = 2–a^3^Π, *v* = 3 band are orders of magnitude larger than those of the nearby d^3^Π, *v* = 3–a^3^Π, *v* = 3 band. If we assume that the ionization efficiencies from the e^3^Δ, *v* = 2 and d^3^Π, *v* = 3 states are the same, we can estimate the intensity ratio of the two bands by comparing the excitation pulse energies required for the same ion signal. This puts the ratio of the band intensities at (2.5 ± 0.5) × 10^–4^.

The calculated lifetime of the e^3^Δ state is 6.0 ns.^4^ Such a lifetime is too short to be accurately determined via time-delayed ionization with laser pulses of 5 ns duration. Our time-delayed ionization measurements confirm that the lifetime of several low-*J* levels in the e^3^Δ, *v* = 2 state is very short, certainly below 10 ns.

### f^3^Σ^+^ State of AlF

**Figure 6:**
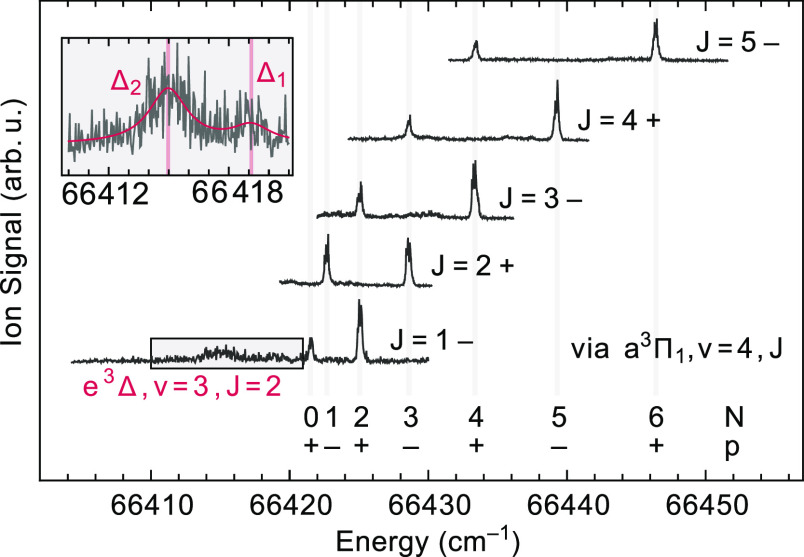
Rotationally resolved spectra to the f^3^Σ^+^, *v* = 1 state recorded via different rotational levels *J* in the a^3^Π_1_, *v* = 4 state. The individual traces are normalized and vertically offset for clarity. The broad structure at around 66,415 cm^–1^ in the lowest trace (shown in the inset) is due to transitions to the two *J* = 2 levels of the predissociated e^3^Δ, *v* = 3 state.

Some 30 cm^–1^ below the e^3^Δ, *v* = 2 – a^3^Π, *v* = 3 band, we observe transitions from the a^3^Π, *v* = 3 state to yet another electronically excited state. This state is readily identified as a ^3^Σ^+^ state as only levels with *N* = *J* ± 1 can be reached when exciting from *J* levels in the a^3^Π_1_ state with parity (−1)^*J*^. We refer to this state as the f^3^Σ^+^, *v* = 0 state, even though this state has previously been documented as the e^3^Σ^+^, *v* = 0 state.^1^ Our measurements reproduce the earlier reported term values, and in addition, we observe the *N* = 0 level. We explicitly verify that there is no lower vibrational level belonging to this f^3^Σ^+^ state, and we do identify its *v* = 1 and *v* = 2 levels. The spectra of the f^3^Σ^+^, *v* = 1–a^3^Π_1_, *v* = 4 band are shown in Figure 6. It is seen that the *R*(*J*) lines are always stronger than the *P*(*J*) lines. A triplet structure is observed on the rotational lines of the f^3^Σ^+^ ←a^3^Π_1_ transition that appears to be similar to that observed on the b^3^Σ^+^ ← a^3^Π transition, where the hyperfine structure has been fully resolved.^7^

The energies and rotational constants that we extracted from these spectra are given in Table 3. In this case, *E*_*v*_ is the energy of the *N* = 0 level in the f^3^Σ^+^, *v* state. It should be noted that the lines to the f^3^Σ^+^, *v* = 2 state appear to be around twice as broad as those to the lower vibrational states, which might be indicative of predissociation.

**Table 3:** Energies *E*_*v*_ and Rotational Constants *B*_*v*_ of the Experimentally Observed Vibrational Levels *v* in the f^3^Σ^+^ State^a^

*v*	*E*_*v*_	*B*_*v*_	σ
0	65,488.17(4)	0.594(1)	0.02
1	66,421.50(6)	0.593(1)	0.03
2	67,343.16(8)	0.582(2)	0.07

a The parameter σ is the standard deviation of the fit. All values are given in cm^–1^.

The broad structure on the baseline that can be observed in several traces shown in Figure 6 is attributed to the residual rotational structure of the e^3^Δ, *v* = 3–a^3^Π_1_, *v* = 4 band. When reducing the intensity of the excitation laser, this broad structure becomes relatively more pronounced, indicating that it originates from a band that is stronger than the f^3^Σ^+^, *v* = 1 – a^3^Π, *v* = 4 band. Based on the extrapolation from the *v* = 0–2 data, the two *J* = 2 levels of the e^3^Δ, *v* = 3 state are expected to be just above 66,420 cm^–1^. Two broadened lines, with the expected spacing of about 4.1 cm^–1^ and with the expected intensity ratio, can be discerned when exciting from the *J* = 1, negative-parity level in the a^3^Π_1_, *v* = 4 state, as shown in the inset to Figure 6. It appears that the rotational levels in the e^3^Δ, *v* = 3 state are downshifted by about 5 cm^–1^ and that the rotational lines exciting these levels are lifetime-broadened to about 1.3 cm^–1^, indicating predissociation on a time-scale of about 4 ps.

We measured the lifetime of several low-*J* levels in the f^3^Σ^+^, *v* = 0 state and found values of (30 ± 6) ns. This is comparable to the lifetime of the d^3^Π state. Both the f^3^Σ^+^ and the d^3^Π states can decay at a significant rate to the c^3^Σ^+^, the b^3^Σ^+^, and the a^3^Π states.

In Table 4, the equilibrium parameters for the electronic potentials of the d^3^Π, e^3^Δ, and f^3^Σ^+^ states of AlF are given, as well as the radiative lifetime τ. The equilibrium internuclear distances *r*_eq_ are the values determined from the equilibrium rotational constants given above, reduced by 0.0027 Å for the d^3^Π state and increased by 0.0027 Å for the e^3^Δ state (see the Section on the perturbation between the e^3^Δ and d^3^Π states).

**Table 4:** Equilibrium Parameters for the Electronic Potentials of the d^3^Π, e^3^Δ, and f^3^Σ^+^ States of AlF^a^

	d^3^Π	e^3^Δ	f^3^Σ^+^
*T*_e_	62,434.6(3)	63,204.5(3)	65,017.1(3)
ω_e_	944.5(2)	941.3(1)	945.0(1)
ω_e_*x*_e_	5.21(4)	6.57(10)	5.84(10)
*B*_e,eff_	0.5931(4)	0.5911(5)	0.599(5)
α_e_	0.0050(1)	0.0045(3)	0.006(3)
*A*_e_	5.73(1)	–0.34(5)	
ζ_e_	–0.024(3)	0.11(3)	
τ (ns)	40(4)	<10	30(6)
*r*_eq_ (Å)	1.5940^b^	1.6021^b^	1.5888
*B*_e,depert_	0.5951^c^	0.5891^c^	

a If not stated otherwise, all values are given in cm^–1^.

b Derived from *B*_e,eff_ after deperturbation.

c Calculated from *r*_eq_.

### X^2^Σ^+^ State of AlF^+^

The ro-vibrational levels of the d^3^Π state are ideally suited as intermediate levels for an accurate measurement of the ionization potential (IP) of AlF. These levels are only one visible photon away from the IP and have a sufficiently long lifetime to enable a well-defined, single-photon ionization experiment. For this, the AlF molecules are prepared in a single ro-vibrational level of the d^3^Π, *v* = 0 state. After a time delay of about 20 ns, the ionization laser is fired to excite the molecules further. The ionization laser is scanned over the region where the ionization onset is expected. Laser preparation and ionization take place in a zero electric field, but to detect the ions, the extraction fields of the mass spectrometer need to be switched on at some point. These extraction fields cause field ionization of laser-prepared, long-lived Rydberg states. Instead of seeing a sharp ionization onset, the onset of ionization will occur gradually with increasing photon energy and begins at energies below what is required to reach the lowest rotational levels in the cation. It is therefore important to wait as long as possible with switching the extraction fields on such that the Rydberg states have decayed. The geometry of our ionization detection region and the beam velocity of about 800 m/s set an upper limit to this wait-time of about 12 μs.

**Figure 7:**
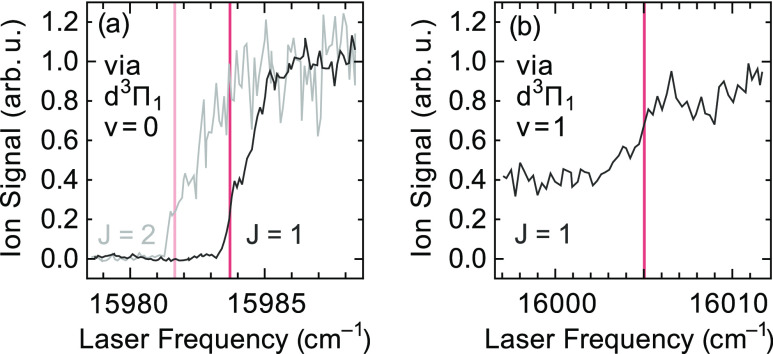
Measurements of the AlF^+^ ion signal versus ionization laser frequency. (a) The onset of ionization from the *J* = 1 (black) and *J* = 2 (gray) levels of the d^3^Π_1_, *v* = 0 state is used to determine the ionization potential of AlF. (b) The increase in the ionization signal starting from the d^3^Π_1_, *v* = 1, *J* = 1 level of AlF occurs as ionization to the first vibrationally excited state in the ion becomes possible.

Measurements of the ion signal as a function of the photon energy of the ionization laser, with a pulsed-field extraction delay of 12 μs, are shown in Figure 7. In Figure 7a, two ionization onset curves, starting from the *J* = 1 level (higher onset) and from the *J* = 2 level (lower onset) of the d^3^Π_1_, *v* = 0 state, are shown. The onsets are seen to occur at frequencies that differ by the energy separation of the starting levels in the d^3^Π state, as expected. Moreover, in both curves, a clear step can be recognized that can be attributed to first reaching only the *N* = 0 level and then, at an energy about 1.2 cm^–1^ higher, also the *N* = 1 level of the X^2^Σ^+^ state of the AlF^+^ cation. From these measurements, the energy of the *N* = 0 level of the X^2^Σ^+^, *v* = 0 state of the AlF^+^ cation is concluded to be at least 78,491 cm^–1^ but not more than 78,493 cm^–1^ above the *N* = 0 level of the X^1^Σ^+^, *v* = 0 state of AlF. It is difficult to define the field-free IP more precisely than this as stray electric fields of 10 mV/cm can already lower the IP by 0.5 cm^–1^. This value of (78,492 ± 1) cm^–1^ for the IP is about 20 cm^–1^ larger, and over 1 order of magnitude more accurate, than the best value reported for this to date.^15^

In Figure 7b, a step in the ionization signal, increasing the ion signal by about a factor of two, is seen in the same spectral region when starting from the *J* = 1 level of the d^3^Π_1_, *v* = 1 state. This step results from reaching the X^2^Σ^+^, *v* = 1 state of the cation. Relative to the onset starting from the *J* = 1 level of the d^3^Π_1_, *v* = 0 state shown in Figure 7a, this step is about (21 ± 2) cm^–1^ higher in energy. This means that ω_e_ – 2ω_e_*x*_e_ in the X^2^Σ^+^ state of the ion is (955 ± 2) cm^–1^, which compares well to the calculated value of 958 cm^–1^.^20^

### Perturbation of the d^3^Π and e^3^Δ States

**Figure 8:**
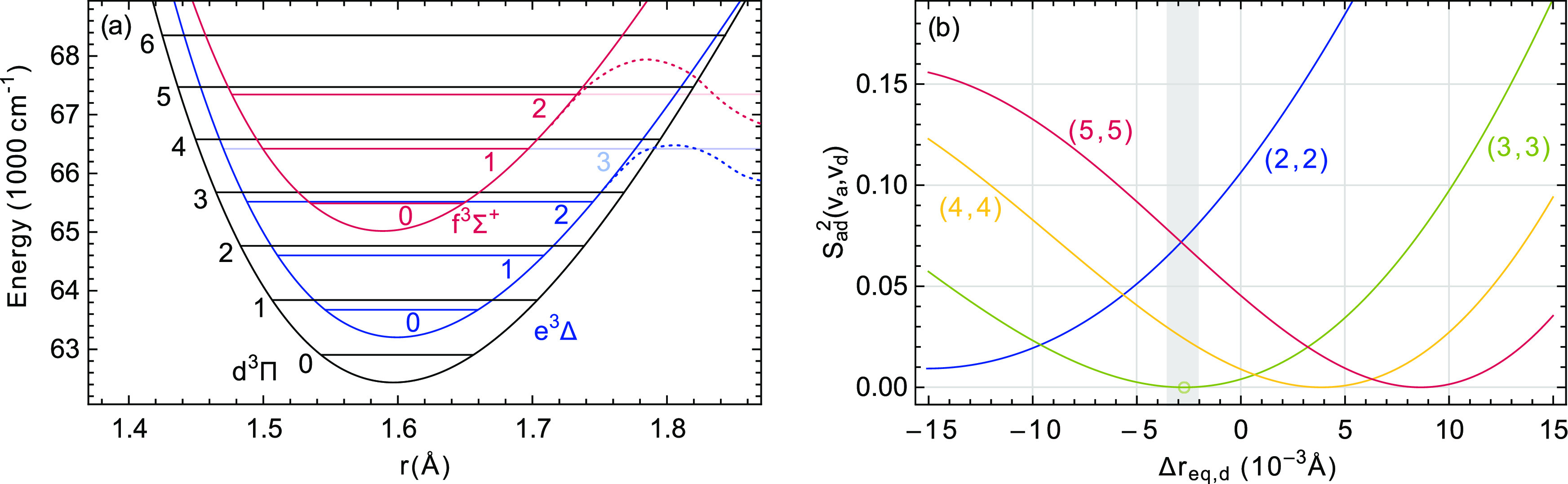
(a) Expanded view of the Morse potentials of the d^3^Π, e^3^Δ, and f^3^Σ^+^ states around their minima. The vibrational levels that have been observed for each of these states are indicated. Dashed lines schematically illustrate potential barriers for the states where predissociation is observed. (b) Calculated Franck–Condon factors  for the diagonal bands of the d^3^Π–a^3^Π transition as a function of Δ*r*_eq,d_, which is the difference of the equilibrium internuclear distance of the d^3^Π state from 1.5967 Å, the value obtained from the effective (perturbed) rotational constant *B*_e,eff_. The shaded bar indicates the range of Δ*r*_eq_ values consistent with our observations of the weak *v* = 3 diagonal band.

To understand the observed, unexpected intensity distribution of the rotational lines in the d^3^Π, *v* = 3–a^3^Π, *v* = 3 band as shown in Figure 4, we investigate the effect of a perturbation of the d^3^Π state and the e^3^Δ state. An interaction between these electronic states is the most obvious one to consider as the d^3^Π, *v* levels are only some 750 cm^–1^ below the e^3^Δ, *v* levels and 160 cm^–1^ above the e^3^Δ, *v*–1 levels. In the following, we show that this perturbation can explain our observations. Moreover, a deperturbation analysis leads to a slight correction of the internuclear potentials of the d^3^Π and e^3^Δ states. In Figure 8a, the Morse potentials of both of these states as well as of the f^3^Σ^+^ state are shown, and the vibrational levels that have been characterized are drawn in. These Morse potentials are derived from the parameters ω_e_, ω_e_*x*_e_, and *r*_eq_, as given in Table 4, with the value of *D*_e_ taken as ω_e_^2^/(4ω_e_*x*_e_).

The perturbation between the e^3^Δ and d^3^Π states can be due to spin–orbit interaction or spin–rotation interaction. The matrix elements for the perturbation between a ^3^Π state and a ^3^Δ state, both described in Hund’s-case (a) bases, depend on the Ω-manifolds of the electronic states, and the only nonzero ones are given by^21^
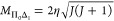
14
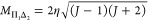
15
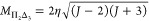
16
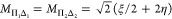
17with the spin–orbit interaction parameter ξ and the spin–rotation interaction parameter η. The interaction between specific vibrational levels *v*_d_ and *v*_e_ is given by the product of the vibrational overlap integral  of those vibrational levels, i.e., by the square root of the Franck–Condon factor, with an overall spin–orbit (*A*_so_) or spin–rotation (*E*_sr_) interaction parameter between the electronic d^3^Π and e^3^Δ states, that is
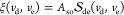
18
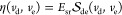
19

The Franck–Condon matrix between the e^3^Δ state and the d^3^Π state is highly diagonal, i.e.,  is close to one when *v*_d_ = *v*_e_ and rapidly decreases with increasing |*v*_d_ – *v*_e_|. From the *B*_e,eff_ values given in Table 4, the internuclear equilibrium distance for the d^3^Π state would be determined as 1.5967(5) Å, only slightly shorter than the equilibrium distance of 1.5994(7) Å for the e^3^Δ state. In particular, the value for the Franck–Condon factor  between the *v*_d_ = 3 and *v*_e_ = 2 levels would be expected to be only 0.009 in that case.

#### Effect on the Line Intensities

The transition intensity (square of the transition dipole moment) of the e^3^Δ−a^3^Π transition has been calculated to be about 20 times larger than that of the d^3^Π–a^3^Π transition.^4^ The Franck–Condon factor for the e^3^Δ, *v* = 2–a^3^Π, *v* = 3 band is about 0.30 (see Table 5), and this band is the likely candidate that the intensity of the d^3^Π, *v* = 3–a^3^Π, *v* = 3 band is borrowed from. As indicated earlier, the strongest lines in the d^3^Π, *v* = 3–a^3^Π, *v* = 3 band are observed to have about (2.5 ± 0.5) × 10^–4^ of the intensity of those in the e^3^Δ, *v* = 2–a^3^Π, *v* = 3 band, and lines with the expected intensity pattern for an allowed d^3^Π–a^3^Π band are not observed at all. Given the signal-to-noise ratio in the spectra shown in Figure 4 of about 25, this implies that the transition intensity of the *v* = 3–*v* = 3 band of the d^3^Π–a^3^Π transition is at least a factor of 10^5^ smaller than that of the e^3^Δ, *v* = 2–a^3^Π, *v* = 3 band. When the transition dipole moment of the d^3^Π–a^3^Π transition would be constant, i.e., independent of the internuclear distance, this would mean that the Franck–Condon factor of this diagonal band is less than 6 × 10^–5^. As the electronic potentials of the e^3^Δ and d^3^Π states are rather similar, the Franck–Condon factor for the diagonal e^3^Δ, *v* = 3–a^3^Π, *v* = 3 band will also be small. Given that the e^3^Δ, *v* = 3 state is predissociated, its vibrational wave function will be distorted. We therefore exclude the e^3^Δ, *v* = 3–a^3^Π, *v* = 3 band when considering the intensity borrowing.

**Table 5:** Franck–Condon Factors , , and  of the a^3^Π–d^3^Π, a^3^Π–e^3^Δ, and a^3^Π–f^3^Σ^+^ Bands, Calculated Using a Morse Potential Derived from the Parameters ω_e_, ω_e_*x*_e_, and *r*_eq_, as Given in Table 4^a^

	*v*_a_ = 0	*v*_a_ = 1	*v*_a_ = 2	*v*_a_ = 3	*v*_a_ = 4	*v*_a_ = 5
*v*_d_ = 0	0.661	0.264	0.062	0.011	0.002	0.000
*v*_d_ = 1	0.282	0.227	0.318	0.132	0.034	0.007
*v*_d_ = 2	0.052	0.369	0.043	0.269	0.182	0.065
*v*_d_ = 3	0.005	0.122	0.347	0.000	0.185	0.204
*v*_d_ = 4	0.000	0.018	0.189	0.275	0.024	0.105
*v*_d_ = 5	0.000	0.001	0.038	0.242	0.190	0.070
*v*_e_ = 0	0.749	0.212	0.035	0.004	0.000	0.000
*v*_e_ = 1	0.222	0.384	0.297	0.081	0.014	0.002
*v*_e_ = 2	0.028	0.331	0.175	0.309	0.125	0.028
*v*_e_ = 3^b^	0.002	0.068	0.370	0.064	0.282	0.159
*v*_f_ = 0	0.611	0.288	0.081	0.017	0.003	0.001
*v*_f_ = 1	0.315	0.159	0.307	0.156	0.049	0.012
*v*_f_ = 2^b^	0.067	0.378	0.013	0.226	0.196	0.086

a For the corresponding values of the a^3^Π state, see ref (6).

b Predissociated.

In Figure 8b, the Franck–Condon factors for several of the diagonal bands of the d^3^Π–a^3^Π transition are shown as a function of the equilibrium internuclear distance of the d^3^Π state. The zero on the horizontal axis corresponds to an equilibrium internuclear distance for the d^3^Π state of 1.5967 Å, which is the value extracted from the equilibrium rotational constant given in Table 4. The internuclear equilibrium distance for the a^3^Π state is accurately known^6^ as *r*_eq,a_ = 1.64708 Å and is kept fixed at this value. When the equilibrium internuclear distance of the d^3^Π state is 1.5967 Å, the Franck–Condon factors for the 3–3 and 4–4 bands are seen to be very similar. Experimentally, both the 2–2 and the 4–4 bands are known to have transition intensities that are orders of magnitude larger than that of the 3–3 band. Even though the dependence of the transition dipole moment on the internuclear distance can influence this somewhat, this indicates that the real equilibrium internuclear distance of the d^3^Π state is about 0.0027 Å smaller than 1.5967 Å.

To quantitatively model the relative line intensities observed in the spectra of the d^3^Π, *v* = 3–a^3^Π, *v* = 3 band, we diagonalize 6 × 6 matrices, set up on a Hund’s-case (a) basis and including the three Ω-manifolds of the two interacting states, for each *J* value. As only transitions to a ^3^Δ_2_ manifold have nonzero Hönl–London factors from a ^3^Π_1_ manifold,^22^ we calculate the fraction of e^3^Δ_2_, *v* = 2 character in the wave function of the d^3^Π_Ω_, *v* = 3 state, denoted as *c*_Δ_2__(d^3^Π_Ω_,*J*). To determine the ξ(3, 2)/η(3, 2) = *A*_so_/*E*_sr_ ratio that reproduces the experimentally observed relative line intensities the best, we define the intensity ratio  as
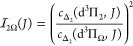
20

**Figure 9:**
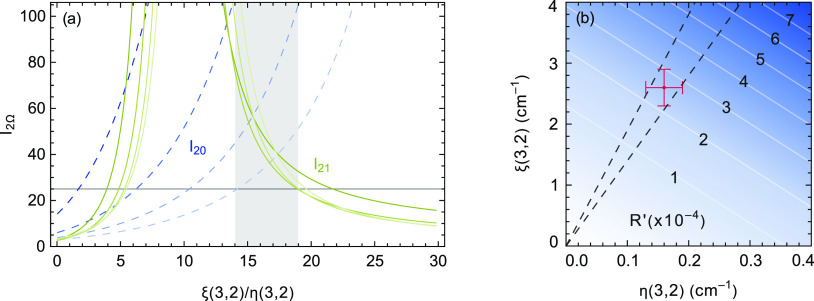
(a)  (solid green lines) and  (dashed blue lines) for *J* = 2–5 (shown with decreasing color saturation), see eq 20. Values within the shaded area (14 < ξ(3, 2)/η(3, 2) < 19) agree with the experimental observation . (b) Ratio of the intensities of the d^3^Π_2_, *v* = 3, *J* = 2 ← a^3^Π_1_, *v* = 3, *J* = 1 and e^3^Δ_2_, *v* = 2, *J* = 2 ← a^3^Π_1_, *v* = 3, *J* = 1 lines that is experimentally found to be (2.5 ± 0.5) × 10^–4^. The area between the dashed lines corresponds to the shaded area in (a). Both criteria are fulfilled for ξ(3, 2) = (2.6 ± 0.3) cm^–1^ and η(3, 2) = (0.16 ± 0.03) cm^–1^, indicated by the red point.

This gives the ratio of the intensities of the lines that start from a certain rotational level in the a^3^Π_1_, *v* = 3 state and that reach levels with the same value of *J* in the d^3^Π_2_, *v* = 3 and in the Ω = 0, 1 manifolds of the d^3^Π_Ω_, *v* = 3 state. A plot of  and  for *J* = 2–5 as a function of the ratio ξ(3, 2)/η(3, 2) is shown in Figure 9a. These curves are independent of the actual values of ξ(3, 2) and η(3, 2) when ξ(3, 2) ≤ 5 cm^–1^ and η(3, 2) ≤ 0.3 cm^–1^. Experimentally, lines to the Ω = 2 manifold are known to basically be the only ones observed, and the value of  and  thus has to be larger than about 25 for *J* = 2–5. For this to hold, it is seen that the ratio of ξ(3, 2)/η(3, 2) has to be between 14 and 19. The signs of ξ(3, 2) and η(3, 2) have to be the same; for negative values of the ξ(3, 2)/η(3, 2) ratio, the conditions on  and  cannot be met.

In Figure 9b, the calculated ratio of the intensities of two lines, , ending up in the *J* = 2 level of the d^3^Π_2_, *v* = 3 state and in the *J* = 2 level in the e^3^Δ_2_, *v* = 2 state is shown. Both lines start from the same *J* = 1 level in the a^3^Π_1_, *v* = 3 state. The intensity ratio is shown as a function of the values of ξ(3, 2) and η(3, 2). The two criteria, namely, that the ξ(3, 2)/η(3, 2) ratio is between 14 and 19 and that the intensity ratio is between 2 × 10^–4^ and 3 × 10^–4^, are fulfilled for ξ(3, 2) = (2.6 ± 0.3) cm^–1^ and η(3, 2) = (0.16 ± 0.03) cm^–1^. The simulated spectrum of Figure 4b is calculated with the thus obtained values for *c*_Δ_2__(d^3^Π_Ω_,*J*), using eq 10. It is seen that in this way, the observed relative line intensities are well reproduced.

#### Effect on the Line Positions

When the strength of the interaction between the e^3^Δ, *v* = 2 and the d^3^Π, *v* = 3 states is on the order of magnitude of several cm^–1^, one would expect to observe distortion of the regular rotational energy level structure. This is particularly so because the strength of the interaction between levels with the same vibrational quantum number *v* in the e^3^Δ and d^3^Π states is larger with the factor , whereas the energy separation of the interacting levels is only more by less than a factor of five. The magnitude of the shift of the energy levels is given by the square of the interaction strength divided by the energy separation, and the direction of the shift is such that the interacting levels repel each other. The downward shift of the ro-vibrational levels in the d^3^Π state due to spin–orbit interaction with levels with the same vibrational quantum number in the e^3^Δ state, for instance, will thus approximately be . This seemed to be a contradiction at first as no shift of the levels or distortion of the regular rotational structure of the levels have been observed, whereas with the expected ratio of  of about 100, these shifts should have been readily detectable.

As the *J* = 0 levels of the d^3^Π state are not influenced by the interaction with the e^3^Δ state, any shift of the energy levels can be recognized most directly when transitions to this *J* = 0 level are included in the spectra. Unfortunately, these transitions cannot be observed in the spectra recorded from the a^3^Π_1_ state, as shown in Figures 3 and 4. When transitions are recorded to the d^3^Π state via the b^3^Σ^+^ state, this lowest rotational level can be reached, as explicitly shown in Figure 4. However, the spectral resolution is, in that case, limited to about 0.3 cm^–1^ due to the span of the hyperfine structure in the b^3^Σ^+^ state, preventing an accurate determination of a possible energy level shift.^7^

From the matrix elements for the perturbation given in eq 14–17, it is seen that for pure Hund’s-case (a) states, the effect of spin–orbit interaction is *J*-independent and absent for Ω = 0 levels. The shift of the levels in the d^3^Π state due to the spin–orbit interaction with the e^3^Δ state will therefore mainly be absorbed in the values of *A*_*v*_ and λ_*v*_ of the d^3^Π state. Both the spin–orbit and the spin–rotation interactions will also influence the term-values *E*_*v*_, but as this effect is expected to be very similar for the lowest four vibrational levels of the d^3^Π state, this will go largely unnoticed. The effect of the spin–rotation interaction will largely be absorbed in the value of the rotational constant. The effective rotational constants for the d^3^Π, *v* = 0–3 levels that are extracted from the spectra will be slightly less than the deperturbed rotational constants, i.e., the values expected purely due to end-over-end rotation. The rotational constant *B*_0_ listed in Table 1, for instance, will be reduced by about (2η(0,0))^2^/(750 cm^–1^) relative to its deperturbed value. The deperturbed equilibrium rotational constant for the d^3^Π state, *B*_e,depert_, will be larger by about this amount than the *B*_e,eff_ value (see Table 4). For the e^3^Δ, *v* = 0 state, the shift of the rotational levels will be equal in magnitude but in the opposite direction, and the *B*_e,depert_ value of the e^3^Δ state is less by (2η(0,0))^2^/(750 cm^–1^) than the *B*_e,eff_ value. As the equilibrium internuclear distances need to be calculated from the *B*_e,depert_ values, *r*_eq_(d^3^Π) will be slightly shorter than the value of 1.5967(5) Å, while *r*_eq_(e^3^Δ) will be the same amount larger than the value of 1.5994(7) Å extracted from the *B*_e,eff_ value given in Table 4.

#### Summary of the Perturbation Analysis

From the observation that the d^3^Π, *v* = 3 – a^3^Π, *v* = 3 band has near-zero transition intensity and from the Franck–Condon factors shown in Figure 8b, it is concluded that the real equilibrium internuclear distance of the d^3^Π state is 0.0027 Å smaller than 1.5967 Å. The unexpected pattern of borrowed intensities is well explained by a perturbation of the d^3^Π state with the e^3^Δ state. This, combined with the equal but opposite energy level shift due to the perturbation, puts the real equilibrium internuclear distance of the e^3^Δ state at a 0.0027 Å larger value than 1.5994 Å. This has a significant effect on the off-diagonal Franck–Condon factors between the d^3^Π state and the e^3^Δ state; as can be seen from Table 6, the value for  is found to be 0.043 instead of 0.009.

**Table 6:** Franck–Condon Factors  between the d^3^Π and e^3^Δ States, Calculated Using Morse Potentials Derived from the Parameters ω_e_, ω_e_*x*_e_, and *r*_eq_, as Given in Table 4

	*v*_d_ = 0	*v*_d_ = 1	*v*_d_ = 2	*v*_d_ = 3	*v*_d_ = 4	*v*_d_ = 5
*v*_e_ = 0	0.988	0.012	0.000	0.000	0.000	0.000
*v*_e_ = 1	0.011	0.963	0.026	0.000	0.000	0.000
*v*_e_ = 2	0.000	0.024	0.932	**0.043**	0.000	0.000
*v*_e_ = 3^a^	0.000	0.001	0.040	0.895	0.064	0.000

a Predissociated.

From the analysis of the line intensities, the values of ξ(3, 2) = (2.6 ± 0.3) cm^–1^ and η(3, 2) = (0.16 ± 0.03) cm^–1^ are found. Together with the value for , this results in values for the overall spin–orbit and spin–rotation interaction parameters between the electronic d^3^Π and e^3^Δ states of *A*_so_ = (12.5 ± 1.5) cm^–1^ and *E*_sr_ = (0.77 ± 0.15) cm^–1^.

It is seen from Table 4 that the deperturbed rotational constants for the d^3^Π state and the e^3^Δ state differ from the effective rotational constants by 0.0020 cm^–1^. It has been argued above that this difference is approximately given by (2η(0,0))^2^/(750 cm^–1^). As the value of  is very close to one, this means that we find in this way that *E*_sr_ is about 0.61 cm^–1^, yielding a fully consistent picture.

## Conclusions

In this study, we have spectroscopically characterized the lowest seven vibrational levels of the newly found d^3^Π state of AlF and we have unambiguously identified the electronic state just above that as a ^3^Δ state. The lowest three vibrational levels of this e^3^Δ state have been characterized, whereas only some broad remnants of the *v* = 3 level could be detected; this vibrational level apparently predissociates on a time scale of a few picoseconds. We have characterized the *v* = 1 and *v* = 2 levels of a yet higher-lying ^3^Σ^+^ state, whose *v* = 0 level had already been reported upon earlier, and this state should be referred to as the f^3^Σ^+^ state from now on. The radiative lifetimes of the d^3^Π and f^3^Σ^+^ states have been found as (40 ± 4) ns and as (30 ± 6) ns, whereas the lifetime of the e^3^Δ state was found to be shorter than 10 ns and, thereby, too short to be measured exactly with the present setup. The experimental data on these three electronic states of AlF are summarized in Table 4. By ionization from the d^3^Π state, the ionization potential of AlF has been accurately determined as (9.73177 ± 0.00012) eV.

A most interesting observation that has been made is that the transition intensity of the d^3^Π, *v* = 3 – a^3^Π, *v* = 3 band is close to zero. This peculiarity of a missing diagonal band might have contributed to the d^3^Π state not having been reported upon earlier. The observation of near-zero transition intensity makes an accurate determination of the difference in equilibrium internuclear distance of the d^3^Π state and the a^3^Π state possible, namely, by evaluating where the Franck–Condon factor for this band goes to zero. It is unlikely that any contribution from the internuclear distance dependence of the transition dipole moment would significantly contribute to the difference in equilibrium bond distance extracted from the intensity analysis presented here. Due to the negligible intensity of the *v* = 3–*v* = 3 band of the d^3^Π–a^3^Π transition, we could recognize the very weak intensity that is borrowed from the nearby and strong e^3^Δ, *v* = 2 – a^3^Π, *v* = 3 band. This in turn enabled a detailed analysis of the spin–orbit and spin–rotation interactions between the d^3^Π state and the e^3^Δ state, from which a spin–orbit interaction parameter *A*_so_ of about 12.5 cm^–1^ and a spin–rotation interaction parameter *E*_sr_ of about 0.8 cm^–1^ have been determined. These parameters, which might have error bars of up to 20%, reproduce the borrowed rotational line intensities very well. The shift of the energy levels caused by the spin–rotation interaction is mainly absorbed into slightly perturbed values of the equilibrium rotational constants in the d^3^Π and e^3^Δ states. After correction for the spin–rotation interaction, the real equilibrium internuclear distances can be extracted from the deperturbed rotational constants, and these agree with those expected from the Franck–Condon analysis.

In their overview paper, Barrow, Klopp, and Malmberg report on the observation of the displacement of a line in the spectrogram of the f^3^Σ^+^, *v* = 0–c^3^Σ^+^, *v* = 0 band.^1^ It appears that the *N* = 41 level of the f^3^Σ^+^, *v* = 0 state is shifted to lower energy by about 0.35(5) cm^–1^, whereas the neighboring levels are hardly affected. From the parameters given in Table 2, we find that the *N* = 41 level [in Hund’s-case (b) notation] of the e^3^Δ, *v* = 2 state is near degenerate with the *N* = 41 level of the f^3^Σ^+^, *v* = 0 state. As the e^3^Δ, *v* = 2 state has some ^3^Π character mixed in, it will be the interaction of these near-degenerate *N* = 41 levels that causes this observed line displacement.

## Supporting Information Available

List of the observed energy levels in the d^3^Π, e^3^Δ, and f^3^Σ^+^ states measured via the d^3^Π ← a^3^Π, e^3^Δ ← a^3^Π, and f^3^Σ^+^ ← a^3^Π bands and wave function characters of the d^3^Π, *v* = 0 and *v* = 3 and e^3^Δ, *v* = 2 states with the squares of these values shown as vertical bars in Figure 3c, 4c, and 5c, respectively (PDF)
