# Supplementary material for: Triplet Rydberg States of Aluminum Monofluoride
Source: J Phys Chem A. 2024 Mar 29;128(14):2752–62. doi: 10.1021/acs.jpca.4c00611 (PMC11017311; doi:10.1021/acs.jpca.4c00611)
Supplement: Supplementary file 1 — jp4c00611_si_001.pdf [file jp4c00611_si_001.pdf]

Triplet Rydberg states of Aluminum Monofluoride

Supporting Information

N. Walter,[a,b] M. Doppelbauer,[a] S. Schaller,[a] X. Liu,[a] R. Thomas,[a] S. Wright,[a] B.G. Sartakov,[a] and G. Meijer[a,c]

[a] Fritz Haber Institute of the Max Planck Society, Faradayweg 4–6, 14195 Berlin, Germany
[b] walter@fhi-berlin.mpg.de, [c] meijer@fhi-berlin.mpg.de

Table S1: Observed energies E_{exp} (in cm^{-1}) of the $\text{d}^3\Pi_{\Omega_\text{d}}, v_\text{d}, J_\text{d}$ levels, reached via the $\text{a}^3\Pi_{\Omega_\text{a}}, v_\text{a}$ states. All quantum numbers are given in the basis of Hund's case (a).

E_{exp}	$E_{\text{exp}} - E_{\text{calc}}$	v_d	Ω_d	J_d	v_a	Ω_a
62901.398	0.003	0	0	1	0	1
62903.277	−0.035	0	0	2	0	1
62906.219	−0.012	0	0	3	0	1
62907.621	−0.024	0	1	1	0	1
62909.695	0.002	0	1	2	0	1
62910.223	0.031	0	0	4	0	1
62912.977	0.009	0	1	3	0	1
62913.891	0.026	0	2	2	0	1
62915.199	−0.029	0	0	5	0	1
62917.555	0.065	0	1	4	0	1
62918.285	−0.025	0	2	3	0	1
62921.418	0.050	0	0	6	0	1
62923.250	0.004	0	1	5	0	1
62923.969	−0.040	0	2	4	0	1
62930.191	−0.034	0	1	6	0	1
62930.973	0.028	0	2	5	0	1
62939.074	−0.018	0	2	6	0	1
63835.234	−0.019	1	0	1	1	1
63837.180	0.027	1	0	2	1	1
63840.051	0.007	1	0	3	1	1
63841.395	−0.026	1	1	1	1	1
63843.527	0.049	1	1	2	1	1
63843.938	−0.025	1	0	4	1	1
63846.770	0.029	1	1	3	1	1
63847.742	0.026	1	2	2	1	1
63848.988	0.033	1	0	5	1	1
63851.164	−0.069	1	1	4	1	1
63852.043	−0.055	1	2	3	1	1
63855.008	−0.033	1	0	6	1	1
63856.949	0.006	1	1	5	1	1
63857.746	0.012	1	2	4	1	1
63863.895	0.032	1	1	6	1	1
63864.598	0.006	1	2	5	1	1
63872.656	−0.001	1	2	6	1	1
64758.895	0.011	2	0	1	2	1
64760.770	0.000	2	0	2	2	1
64763.648	0.008	2	0	3	2	1
64765.117	0.046	2	1	1	2	1
64767.078	−0.044	2	1	2	2	1
64767.520	−0.015	2	0	4	2	1
64770.340	−0.023	2	1	3	2	1

Table S1 Continued from previous column.

E_{exp}	$E_{\text{exp}} - E_{\text{calc}}$	v_{d}	Ω_{d}	J_{d}	v_{a}	Ω_{a}
64771.379	−0.030	2	2	2	2	1
64772.465	−0.021	2	0	5	2	1
64774.809	−0.008	2	1	4	2	1
64775.781	0.046	2	2	3	2	1
64778.512	−0.010	2	0	6	2	1
64780.520	0.043	2	1	5	2	1
64781.332	0.025	2	2	4	2	1
64787.371	0.038	2	1	6	2	1
64788.027	−0.070	2	2	5	2	1
64796.086	0.003	2	2	6	2	1
65672.359	0.005	3	0	1	2	1
65674.188	−0.037	3	0	2	2	1
65677.047	−0.034	3	0	3	2	1
65678.438	−0.051	3	1	1	2	1
65680.609	0.064	3	1	2	2	1
65681.000	0.053	3	0	4	2	1
65683.789	0.016	3	1	3	2	1
65684.898	0.022	3	2	2	2	1
65685.867	0.004	3	0	5	2	1
65688.180	−0.024	3	1	4	2	1
65689.148	−0.015	3	2	3	2	1
65691.891	0.036	3	0	6	2	1
65693.820	−0.013	3	1	5	2	1
65694.695	0.010	3	2	4	2	1
65700.625	−0.020	3	1	6	2	1
65701.398	−0.024	3	2	5	2	1
65709.352	0.008	3	2	6	2	1
66575.156	0.013	4	0	1	4	1
66576.984	−0.020	4	0	2	4	1
66579.844	0.002	4	0	3	4	1
66581.328	0.009	4	1	1	4	1
66583.367	0.009	4	1	2	4	1
66583.672	−0.012	4	0	4	4	1
66586.531	−0.033	4	1	3	4	1
66587.703	0.005	4	2	2	4	1
66588.570	0.002	4	0	5	4	1
66590.969	0.008	4	1	4	4	1
66591.938	−0.005	4	2	3	4	1
66594.539	0.016	4	0	6	4	1
66596.531	−0.006	4	1	5	4	1
66597.445	0.023	4	2	4	4	1
66603.312	0.021	4	1	6	4	1
66604.078	−0.018	4	2	5	4	1

Table S1 Continued from previous column.

E_{exp}	$E_{exp} - E_{calc}$	v_d	Ω_d	J_d	v_a	Ω_a
66611.938	−0.015	4	2	6	4	1
67469.461	0.049	5	0	2	5	1
67472.180	−0.055	5	0	3	5	1
67473.836	−0.009	5	1	1	5	1
67475.844	−0.006	5	1	2	5	1
67476.117	0.064	5	0	4	5	1
67479.008	0.006	5	1	3	5	1
67480.062	−0.049	5	2	2	5	1
67480.898	−0.008	5	0	5	5	1
67483.344	0.001	5	1	4	5	1
67484.344	0.023	5	2	3	5	1
67486.734	−0.089	5	0	6	5	1
67488.953	0.096	5	1	5	5	1
67489.727	−0.026	5	2	4	5	1
67495.508	−0.034	5	1	6	5	1
67496.398	0.025	5	2	5	5	1
67504.172	0.013	5	2	6	5	1
68351.08	−0.03	6	0	2	6	1
68353.80	−0.11	6	0	3	6	1
68355.57	0.05	6	1	1	6	1
68357.47	−0.06	6	1	2	6	1
68357.75	0.06	6	0	4	6	1
68360.59	−0.07	6	1	3	6	1
68361.97	0.08	6	2	2	6	1
68362.43	−0.07	6	0	5	6	1
68365.01	0.03	6	1	4	6	1
68365.98	−0.06	6	2	3	6	1
68368.57	0.22	6	0	6	6	1
68370.52	0.08	6	1	5	6	1
68371.41	0.02	6	2	4	6	1
68376.98	−0.08	6	1	6	6	1
68378.01	0.08	6	2	5	6	1
68385.50	−0.13	6	2	6	6	1

Table S2: Observed energies E_{exp} (in cm^{-1}) of the $e^3\Delta_{\Omega_e}, v_e, J_e$ levels, reached via the $a^3\Pi_{\Omega_a}, v_a$ states. All quantum numbers are given in the basis of Hund's case (a).

E_{exp}	$E_{exp} - E_{calc}$	v_e	Ω_e	J_e	v_a	Ω_a
63674.125	0.020	0	3	3	1	1
63674.945	0.018	0	2	2	1	1
63675.473	−0.031	0	1	1	1	0

Table S2 Continued from previous column.

E_{exp}	$E_{exp} - E_{calc}$	v_e	Ω_e	J_e	v_a	Ω_a
63677.766	−0.003	0	3	4	1	1
63678.348	−0.024	0	2	3	1	1
63678.836	0.028	0	1	2	1	1
63682.566	0.001	0	3	5	1	1
63683.023	−0.010	0	2	4	1	1
63683.387	0.001	0	1	3	1	1
63688.512	−0.004	0	3	6	1	1
63688.883	−0.010	0	2	5	1	1
63689.215	0.022	0	1	4	1	1
63695.891	−0.049	0	2	6	1	1
63696.223	0.024	0	1	5	1	1
63704.418	0.016	0	1	6	1	1
64602.156	−0.031	1	3	3	2	1
64603.141	0.014	1	2	2	2	1
64603.793	0.001	1	1	1	2	0
64605.887	0.048	1	3	4	2	1
64606.500	−0.033	1	2	3	2	1
64607.031	−0.008	1	1	2	2	1
64610.598	−0.013	1	3	5	2	1
64611.137	−0.016	1	2	4	2	1
64611.582	0.021	1	1	3	2	1
64616.547	0.029	1	3	6	2	1
64616.953	−0.006	1	2	5	2	1
64617.328	0.020	1	1	4	2	1
64623.922	−0.026	1	2	6	2	1
64624.254	0.005	1	1	5	2	1
64632.371	−0.004	1	1	6	2	1
65516.965	0.010	2	3	3	3	1
65518.188	−0.026	2	2	2	3	1
65519.098	−0.026	2	1	1	3	0
65520.684	0.053	2	3	4	3	1
65521.527	−0.052	2	2	3	3	1
65522.277	0.004	2	1	2	3	1
65525.406	0.007	2	3	5	3	1
65526.199	0.048	2	2	4	3	1
65526.734	0.017	2	1	3	3	1
65531.242	−0.046	2	3	6	3	1
65531.875	−0.030	2	2	5	3	1
65532.465	0.079	2	1	4	3	1
65538.766	−0.062	2	2	6	3	1
65539.250	0.004	2	1	5	3	1
65547.320	0.020	2	1	6	3	1

Table S3: Observed energies E_{exp} (in cm^{-1}) of the $\mathrm{f}^3\Sigma^+, v_\mathrm{f}, N_\mathrm{f}, p_\mathrm{f}$ levels, reached via the $\mathrm{a}^3\Pi_{\Omega_\mathrm{a}}, v_\mathrm{a}$ states. E_{exp} is the absolute energy of the reached level. The quantum numbers of the $\mathrm{f}^3\Sigma^+$ state are given in the basis of Hund's case (b), the quantum numbers of the $\mathrm{a}^3\Pi$ state in the basis of Hund's case (a).

E_{exp}	$E_{\mathrm{exp}} - E_{\mathrm{calc}}$	v_f	N_f	p_f	v_a	Ω_a
65488.176	0.002	0	0	+	3	1
65489.365	0.002	0	1	−	3	1
65491.717	−0.023	0	2	+	3	1
65495.341	0.035	0	3	−	3	1
65500.039	−0.022	0	4	+	3	1
65506.009	0.005	0	5	−	3	1
65513.136	−0.000	0	6	+	3	1
66421.520	0.020	1	0	+	4	1
66422.674	−0.011	1	1	−	4	1
66425.027	−0.028	1	2	+	4	1
66428.579	−0.033	1	3	−	4	1
66433.412	0.057	1	4	+	4	1
66439.310	0.028	1	5	−	4	1
66446.362	−0.033	1	6	+	4	1
67343.200	0.042	2	0	+	2	4
67344.260	−0.062	2	1	−	4	1
67346.580	−0.070	2	2	+	4	1
67350.230	0.089	2	3	−	4	1
67354.770	−0.026	2	4	+	4	1
67360.710	0.095	2	5	−	4	1
67367.530	−0.068	2	6	+	4	1

Table S4: Wavefunction characters of the $\mathrm{d}^3\Pi, v = 0$, $v = 3$ and $\mathrm{e}^3\Delta, v = 2$ states.

state	v	Ω	J	c_{Π_0}	c_{Π_1}	c_{Π_2}	c_{Δ_1}	c_{Δ_2}	c_{Δ_3}
$\mathrm{d}^3\Pi$	0	0	0	1.000	0.000	0.000	−	−	−
$\mathrm{d}^3\Pi$	0	0	1	0.981	−0.193	0.000	−	−	−
$\mathrm{d}^3\Pi$	0	0	2	0.948	−0.314	0.054	−	−	−
$\mathrm{d}^3\Pi$	0	0	3	0.907	−0.409	0.104	−	−	−
$\mathrm{d}^3\Pi$	0	1	1	0.193	0.981	0.000	−	−	−
$\mathrm{d}^3\Pi$	0	1	2	0.307	0.855	−0.418	−	−	−
$\mathrm{d}^3\Pi$	0	0	4	0.864	−0.480	0.152	−	−	−
$\mathrm{d}^3\Pi$	0	1	3	0.398	0.746	−0.535	−	−	−
$\mathrm{d}^3\Pi$	0	2	2	0.086	0.413	0.907	−	−	−
$\mathrm{d}^3\Pi$	0	0	5	−0.825	0.531	−0.195	−	−	−
$\mathrm{d}^3\Pi$	0	1	4	0.468	0.653	−0.595	−	−	−
$\mathrm{d}^3\Pi$	0	2	3	0.141	0.526	0.839	−	−	−
$\mathrm{d}^3\Pi$	0	0	6	0.790	−0.568	0.231	−	−	−

Table S4 Continued from previous column.

state	v	Ω	J	c_{Π_0}	c_{Π_1}	c_{Π_2}	c_{Δ_1}	c_{Δ_2}	c_{Δ_3}
$d^3\Pi$	0	1	5	−0.520	−0.576	0.630	–	–	–
$d^3\Pi$	0	2	4	0.186	0.586	0.789	–	–	–
$d^3\Pi$	0	0	7	0.760	−0.595	0.261	–	–	–
$d^3\Pi$	0	1	6	0.559	0.512	−0.652	–	–	–
$d^3\Pi$	0	2	5	0.223	0.621	0.751	–	–	–
$d^3\Pi$	0	0	8	0.735	−0.615	0.286	–	–	–
$d^3\Pi$	0	1	7	−0.588	−0.459	0.666	–	–	–
$d^3\Pi$	0	2	6	0.252	0.644	0.722	–	–	–
$d^3\Pi$	3	0	0	1.000	0.000	0.000	0.000	0.000	0.000
$d^3\Pi$	3	0	1	0.982	−0.188	0.000	−0.001	0.000	0.000
$d^3\Pi$	3	1	1	0.188	0.982	0.000	−0.006	0.000	0.000
$d^3\Pi$	3	0	2	0.950	−0.307	0.050	−0.001	0.000	0.000
$d^3\Pi$	3	1	2	0.301	0.867	−0.397	−0.006	0.000	0.000
$d^3\Pi$	3	2	2	−0.078	−0.393	−0.916	0.003	0.006	0.000
$d^3\Pi$	3	0	3	0.911	−0.401	0.098	−0.002	0.001	0.000
$d^3\Pi$	3	1	3	−0.390	−0.762	0.517	0.006	0.000	−0.002
$d^3\Pi$	3	2	3	0.132	0.509	0.851	−0.004	−0.007	−0.003
$d^3\Pi$	3	0	4	0.870	−0.471	0.144	−0.002	0.002	−0.001
$d^3\Pi$	3	1	4	−0.460	−0.671	0.581	0.007	0.000	−0.003
$d^3\Pi$	3	2	4	0.177	0.572	0.801	−0.005	−0.008	−0.004
$d^3\Pi$	3	0	5	0.831	−0.524	0.186	−0.003	0.002	−0.001
$d^3\Pi$	3	1	5	−0.513	−0.594	0.619	0.007	0.000	−0.004
$d^3\Pi$	3	2	5	0.213	0.610	0.763	−0.005	−0.009	−0.005
$d^3\Pi$	3	0	6	0.797	−0.562	0.222	−0.003	0.003	−0.002
$d^3\Pi$	3	1	6	−0.553	−0.530	0.643	0.008	0.000	−0.005
$d^3\Pi$	3	2	6	0.244	0.635	0.733	−0.006	−0.009	−0.005
$e^3\Delta$	2	3	3	–	–	–	0.126	−0.388	0.913
$e^3\Delta$	2	2	2	–	–	–	−0.453	0.892	0.000
$e^3\Delta$	2	1	1	–	–	–	1.000	0.000	0.000
$e^3\Delta$	2	3	4	–	–	–	0.195	−0.500	0.844
$e^3\Delta$	2	2	3	–	–	–	−0.565	0.728	0.388
$e^3\Delta$	2	1	2	–	–	–	0.892	0.453	0.000
$e^3\Delta$	2	3	5	–	–	–	0.244	−0.560	0.791
$e^3\Delta$	2	2	4	–	–	–	0.618	−0.606	−0.501
$e^3\Delta$	2	1	3	–	–	–	0.815	0.565	0.127
$e^3\Delta$	2	3	6	–	–	–	−0.281	0.597	−0.751
$e^3\Delta$	2	2	5	–	–	–	−0.646	0.514	0.564
$e^3\Delta$	2	1	4	–	–	–	0.762	0.619	0.191
$e^3\Delta$	2	3	7	–	–	–	0.309	−0.621	0.720
$e^3\Delta$	2	2	6	–	–	–	−0.663	0.445	0.602
$e^3\Delta$	2	1	5	–	–	–	0.723	0.649	0.237
$e^3\Delta$	2	3	8	–	–	–	−0.331	0.638	−0.696
$e^3\Delta$	2	2	7	–	–	–	0.674	−0.391	−0.626

Table S4 Continued from previous column.

state	v	Ω	J	c_{Π_0}	c_{Π_1}	c_{Π_2}	c_{Δ_1}	c_{Δ_2}	c_{Δ_3}
$e^3\Delta$	2	1	6	–	–	–	0.694	0.667	0.271
